# Women’s sleep position during pregnancy in low- and middle-income countries: a systematic review

**DOI:** 10.1186/s12978-021-01106-x

**Published:** 2021-03-01

**Authors:** Shania K. Rossiter, Samia Aziz, Alyce N. Wilson, Liz Comrie-Thomson, Tomasina Stacey, Caroline S. E. Homer, Joshua P. Vogel

**Affiliations:** 1 Maternal, Child and Adolescent Health Program, Burnet Institute, 85 Commercial Road, Melbourne, 3000 Australia; 2 School of Human and Health Sciences, University of Huddersfield, Huddersfield, UK

**Keywords:** Sleep position, Pregnancy, Stillbirth, Low- and middle-income countries

## Abstract

**Background:**

Approximately 2.6 million babies are stillborn each year globally, of which 98% occur in low- and middle-income countries (LMICs). A 2019 individual participant data meta-analysis of 6 studies from high-income countries found that maternal supine going-to-sleep position increased the risk of stillbirth. It is not clear whether this impact would be the same in LMICs, and the normal sleep behaviour of pregnant women in LMICs is not well understood.

**Objective:**

Determine the prevalence of different sleeping positions among pregnant women in LMICs, and what (if any) positions were associated with stillbirth using a systematic review.

**Search strategy:**

We systematically searched the databases Medline, Embase, Emcare, CINAHL and Global Index Medicus for relevant studies, with no date or language restrictions on 4 April 2020. Reference lists of included studies were also screened.

**Selection criteria:**

Observational studies of maternal sleep position during pregnancy in LMICs

**Data collection and analysis:**

Recovered citations were screened and eligible studies were included for extraction. These steps were performed by two independent reviewers. Risk of bias was assessed using the Newcastle–Ottawa Scale.

**Main results:**

A total of 3480 citations were screened but only two studies met the inclusion criteria. The studies were conducted in Ghana and India and reported on different maternal sleep positions: supine and left lateral. In Ghana, a prevalence of 9.7% for supine sleeping position amongst 220 women was found. The primary outcome could not be extracted from the Indian study as sleep position information was only reported for women who had a stillbirth (100 of the 300 participants).

**Conclusion:**

There is limited information on maternal sleeping position in LMICs. Since sleep position may be a modifiable risk factor for stillbirth, there is a need for further research to understand the sleep practices and behaviours of pregnant women in LMICs.

*PROSPERO registration:* CRD42020173314

## Plain english summary

In 2015, there were an estimated 2.6 million stillborn babies worldwide, and 98% of these babies were born in LMICs. In order to decrease the global incidence of stillbirth there has been recent interest in investigating aspects of maternal lifestyle during pregnancy that may be associated with an increased risk of stillbirth. One particular aspect of interest is maternal sleep position. There have been two recent systematic reviews that have evaluated the impact of maternal sleep on fetal outcomes and investigated the effect of maternal sleep positions on stillbirth. Both reviews found that maternal supine sleep position was associated with an increased risk of late stillbirth. However, the vast majority of included studies were conducted in HICs indicating that the normal sleep behaviour of pregnant women in LMICs is not well understood.

We aimed to conduct a systematic review to determine the prevalence of different sleep positions during pregnancy amongst pregnant women in LMICs and investigate if these positions were associated with stillbirth. In order to conduct this systematic review, we created a search strategy to systematically search online databases for observational studies of maternal sleep position during pregnancy in LMICs. We searched five databases on 4 April 2020, these were: Medline, Embase, Emcare, CINAHL and Global Index Medicus. Reference lists of included studies were also screened. The recovered citations were screened in duplicate and studies deemed eligible by two independent reviewers were included for extraction. The risk of bias of the included studies was assessed using NOS.

## Background

In 2015, there was an estimated 2.6 million late (at or after 28 weeks’ pregnancy) stillbirths worldwide, which equates to more than 7178 deaths per day [1]. This makes stillbirth the fifth leading cause of death globally; surpassing HIV/AIDS, road traffic accidents and any type of cancer [2]. The majority of stillborn babies are born in low- and middle-income countries (LMICs)—an estimated 98% of all cases [1]. In order to decrease the global incidence of stillbirth, it is critical to identify innovative, effective and low-cost strategies to prevent stillbirth. Interest in modifying maternal sleep position during pregnancy followed the publication of a novel study by Stacey et al. in 2011 [3]. This study, undertaken in New Zealand, found an association between maternal supine sleep position during pregnancy and an increased risk of stillbirth.

The association of maternal supine sleep position with an increased risk of stillbirth is biologically plausible. As pregnancy progresses there is increased aortic and inferior vena caval compression by the gravid uterus [4]. In the supine position this compression is exacerbated and can result in up to an 85% decrease in blood flow through the woman’s inferior vena cava and up to a 30% decrease through the aorta [5–7]. This can in turn lead to a decrease in maternal cardiac output and stroke volume, reducing perfusion of the placental and fetal circulation [8–11]. This can decrease fetal oxygenation and may compromise fetal wellbeing [5, 11, 12].

Similar findings to Stacey et al. have been observed in other studies with similar methodologies and similar effect sizes conducted in high-income countries (HICs) [3, 13–15]. As a result, going-to-sleep on the side positions from 28 weeks of pregnancy are now recommended in Australia, New Zealand and the United Kingdom and have been targeted through stillbirth prevention campaigns [16–18]. There have also been two recent systematic reviews—a 2018 scoping review by Warland et al. and a 2019 individual participant data (IPD) meta-analysis by Cronin et al. that have evaluated the impact of maternal sleep on fetal outcomes [19, 20]. Both reviews found an association between supine sleep position and stillbirth, however the IPD meta-analysis did not include any studies from LMICs, as no studies met the inclusion criteria. The findings from these reviews are of interest because maternal sleep behaviours are potentially modifiable, even in low-resource settings [21]. However, since the vast majority of stillbirths occur in LMICs it is important that maternal sleep behaviours in LMICs be evaluated. We also chose to examine the association in studies from LMICs only, as there is considerable variation in what constitutes normal sleep practices between countries and cultures [22–25]. This systematic review aims to fill this knowledge gap by summarizing the available data on the prevalence of maternal sleep positions during pregnancy in LMICs, and assessing whether there is an association between maternal sleep positions and stillbirth among women in LMICs.

## Methods

This systematic review was conducted in accordance with PRISMA guidelines (see Additional file 1: for PRISMA checklist), and PROSPERO registration number is: CRD42020173314 [26].

### Eligibility criteria

For this systematic review, eligible studies were those reporting prevalence of maternal sleeping position during pregnancy in LMICs (as defined using the World Bank classification for 2021) [27]. Peer-reviewed, non-randomised studies (including observational and cross-sectional studies) were eligible for inclusion. The population of interest was pregnant women from LMICs, regardless of maternal age, gestation, singleton or multiple pregnancy or risk of pregnancy complications.

### Literature searching, data collection and analysis

We searched Medline, Global Index Medicus, Embase, Emcare and CINAHL for relevant studies with no date or language restrictions, and excluded animal studies (see Additional file 2: for search strategy). Additionally, the reference lists of included studies were also screened. All recovered citations were imported into Covidence and duplicates were automatically removed [28] Two authors independently screened titles and abstracts of all identified citations for eligibility, followed by the full-texts. Data were then extracted from the eligible studies and risk of bias was assessed using the Newcastle Ottawa-Scale (NOS).

We extracted data including study design, setting, location, population, and prevalence of maternal sleeping positions using a pre-designed tool (Additional file 3). Data were extracted separately by each reviewer, with results compared to identify differences which were resolved through discussion or consulting a third, more senior, reviewer. Insufficient data were identified to perform a meta-analysis and all data were reported descriptively. Future updates of this review may identify further data, in which case a meta-analysis can be performed. To assess the risk of bias for included studies we used the NOS tool for cohort and case–control studies, and an adapted NOS tool for cross-sectional studies [29, 30].

## Results

**Fig. 1:**
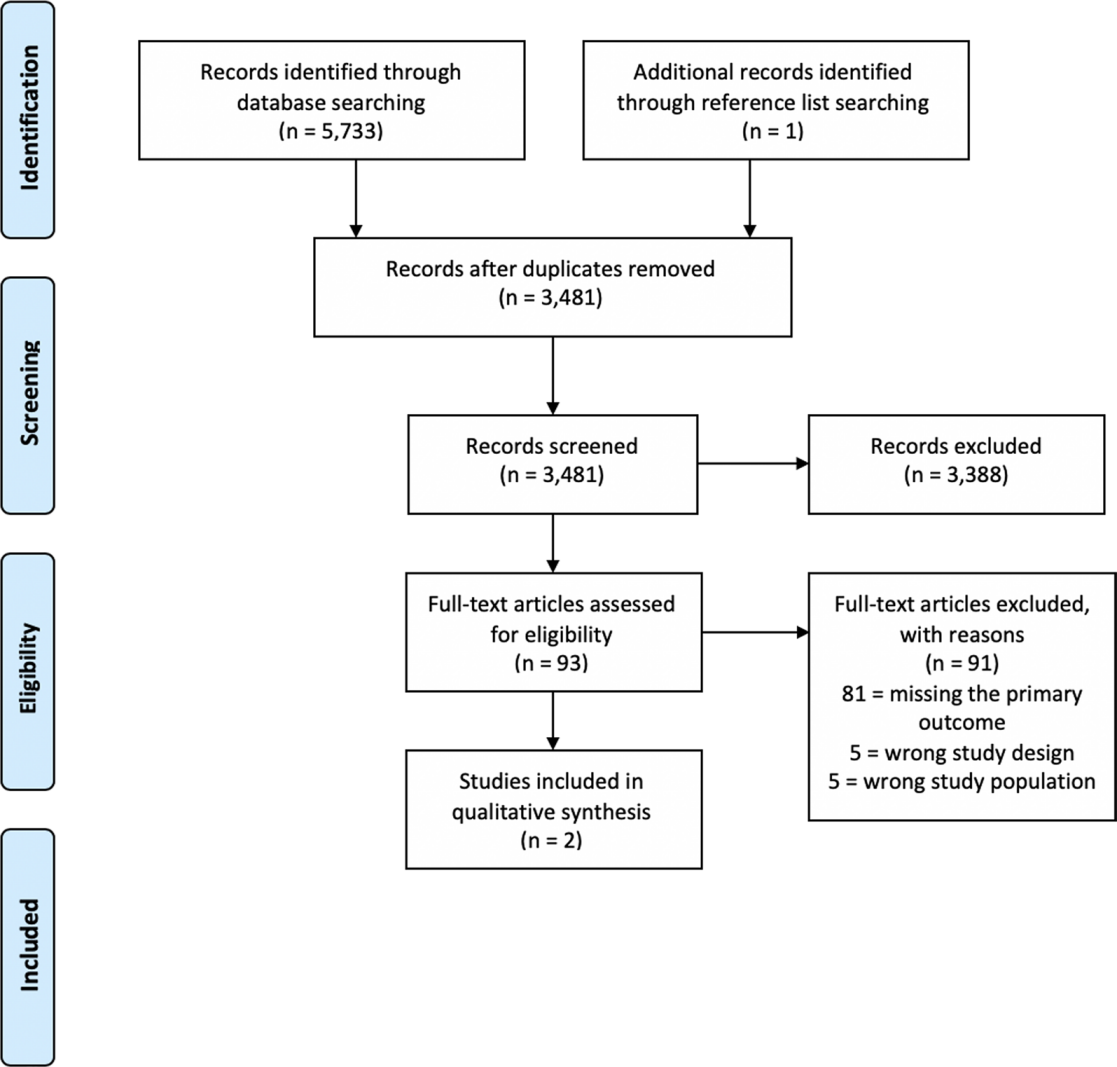
PRISMA flow diagram showing the selection of eligible studies

A total of 5733 citations were identified through the search, and one unique citation through reference list screening. After duplicates were removed, 3481 unique citations remained (Fig. 1). In total, 3388 citations were excluded during the title and abstract screening and 93 full-text articles were reviewed. Of these, two citations were eligible [31, 32]. The remaining studies were excluded due to not reporting the primary review outcome of interest (81 citations), ineligible study design (5 citations) or ineligible study population (5 citations).

The two eligible studies were a 2013 cross-sectional study conducted in Ghana, and a 2017 case–control study conducted in India [31, 32]. Table 1 describes the study level characteristics of the included studies, and Table 2 reports the differences in maternal and pregnancy characteristics and sleep practices between study participants, though both studies provided limited demographic data. The risk of bias of both studies as per NOS assessment was high (score of one) for the Lakshmi et al. study and moderate (score of six) for the Owusu et al. study (Table 3).

**Table 1:** Characteristics of included studies

Study level characteristics	Owusu et al. [31]	Lakshmi et al. [32]
Location	Accra, Ghana	Trivandrum, India
Length of study	June 2011–July 2011	March 2014–September 2015
Study design	Retrospective hospital-based cross-sectional study	Hospital-based case–control study
Population	Postpartum women; 0–48 h postpartum	Postpartum women
Main outcome measure	To investigate the prevalence of sleep disruption and sleep practices among Ghanaian women and to investigate its association with maternal and neonatal outcomes	To identify any possible modifiable risk factors and reduce stillbirth rate on a long-term perspective
Description of sleep position	Most common sleep position during pregnancy	Not defined
Data collection—sleep position	Self-reported	Self-reported
Data collection—stillbirth information	Clinical notes	Hospital data

**Table 2:** Participant level characteristics and going-to-sleep position

Characteristics	Owusu et al. [31]	Lakshmi et al. [32]
Total participants	220	Case (participants who had a stillbirth) n = 100 Control (participants who had a live birth) n = 200 Total = 300
Nationality	Ghanaian	Indian
Age—mean (SD)	29 (5.7) years	Not reported
Age range	Not reported	18–40 years
Parity—n (%) Nulliparous 1–2 3–4 ≥ 5	71 (32.3) 113 (51.4) 31 (14.1) 5 (2.3)	Not reported
Sleep position – n (%) Supine Non-supine	21 (9.7) 195 (90.3)	Not reported Not reported
Stillbirth	9 (4.1)	100 (percentage could not be extracted due to study design)*

Data are number (percentage) or mean (standard deviation)

*Cases were identified as women who had a stillbirth, therefore the prevalence of stillbirth amongst participants could not be extracted

**Table 3:** Newcastle–Ottawa Scale (NOS) scores for the included studies

	Selection score	Comparability score	Exposure/Outcome score	Overall score
Owusu et al. 2013	2/5	2/2	2/3	6/10
Lakshmi et al. 2017	1/4	0/2	0/3	1/9

NOS adapted for cross-sectional studies was used for the Owusu et al. study

Maximum scores: selection = 5, comparability = 2, outcome = 3, and overall = 10

High risk of bias: score ≤ 3

Medium risk of bias: score 4–7

Low risk of bias: score ≥ 8

The traditional NOS tool was used for the Lakshmi et al. study

Maximum scores: selection = 4, comparability = 2, exposure = 3 and overall = 9

High risk of bias: score ≤ 3

Medium risk of bias: score 4–6

Low risk of bias: score ≥ 7

The cross-sectional study by Owusu et al. with a sample size of 220 postpartum women found that 9.7% of participants reported supine sleep position. The remaining 90.3% had varied lateral sleep positions. The prevalence of stillbirth among participants was 4.09%. Owusu et al. found an increase in the odds of stillbirth associated with supine sleeping position compared to non-supine sleeping positions, reporting an odds ratio of 8.00, 95% CI 1.50–43.20, p = 0.016.

The 2017 case–control study by Lakshmi et al. aimed to identify possible modifiable risk factors for stillbirth. An included risk factor was maternal sleep position, so the study reported the prevalence of maternal left lateral sleep position during pregnancy. Due to the study design, sleep position data were only reported from participants who had a stillbirth, the cases in this case–control study. Sleep position data was not collected for the controls, women who had a live birth. Therefore, the prevalence of left lateral sleeping position during pregnancy among all women could not be extracted and the risk of stillbirth associated with sleep position could not be calculated. Among women who experienced stillbirth, Lakshmi et al. found an increase in the odds of non-left lateral sleeping positions compared to a left lateral sleeping position during pregnancy (OR 2.27, 95% CI 1.37–3.76, p < 0.001).

The risk of bias of studies as per NOS assessment was considered to be high for the Lakshmi et al. study and moderate for the Owusu et al. study (Table 3). Publication bias could not be assessed due to the small number of eligible studies.

## Discussion

### Main findings

This systematic review identified very limited data on maternal sleep position during pregnancy in LMICs, and it is currently not possible to draw conclusions. Two eligible studies were identified and reported on different sleep positions; supine and left lateral [31, 32]. In Ghana, Owusu et al. found that women who reported they most commonly slept in a supine sleep position in pregnancy were 8.0 times more likely to experience stillbirth than women who did not (OR 8.00, 95% CI 1.50–43.20, *p* = 0.016) [31]. In India, Lakshmi et al. reported women who had experienced stillbirth were 2.3 times more likely to report sleeping in a non-left lateral sleep position, compared to a left lateral sleep position (OR 2.27, 95% CI 1.37–3.76, p < 0.00) [32]. Overall, this systematic review has demonstrated that the sleep behaviours of women in LMICs during pregnancy are not well documented, despite the established association between maternal sleep position and stillbirth. However, the limited information available aligns with existing evidence from HICs that maternal going-to-sleep position is associated with an increased risk of stillbirth [19]. The very limited available information also suggests that the effect size may be consistent with those from HICs, though this might change with additional evidence. There remains a need for further high-quality research to investigate maternal sleep practices and behaviours in pregnant women from LMICs.

### Strengths and limitations

This systematic review has some limitations. Firstly, although a comprehensive search was undertaken, only peer-reviewed studies were eligible. There is a possibility that eligible studies were not found in the search, although we consider this to be unlikely because reference list screening was performed in conjunction with a broad search strategy. Second, a meta-analysis could not be performed due to differences in outcome reporting between the two eligible studies. Third, the findings of this systematic review are based on two studies with small sample sizes. Both studies were also conducted in a hospital setting so the samples are not representative of all pregnant women in either country, or in LMIC settings broadly, given the high proportion of women who do not attend hospital during pregnancy. Both included studies were reliant on the postpartum participants recalling their sleep position during pregnancy. The participants stillbirth status may have also influenced the accuracy of recall. It is possible that the experience of the stillbirth may have influenced the accuracy of recall of maternal sleep position [33, 34]. If families know that supine position is associated with an increased risk then this may alter their recollection; though whether this experience improves or worsens recall of sleep position is not yet known. Another limitation is that there are differing definitions of sleep position [13]. Furthermore, the gestational age at which sleep position was measured varied between the included studies. Should advice on sleep position be incorporated into antenatal care more widely, the optimal timing of this intervention and how best to communicate it to pregnant women and healthcare providers needs to be carefully considered. A strength of our systematic review is that a broad search strategy was conducted across multiple databases and reference lists were searched for additional studies. Also, the search strategy had no limitations on language or publication date.

### Interpretation

Two recent publications, a scoping review and IPD meta-analysis, have reviewed the evidence regarding supine sleep position and stillbirth [19, 20]. The IPD meta-analysis reported on all available, eligible studies on the topic. The 2013 study from Ghana by Owusu et al. was not included in that review due to ineligibility of study design. Both of the eligible studies for this review were included in the meta-analysis of the scoping review but the odds ratio estimate is not reliable due to the heterogeneous data Warland et al. used [19, 20]. The findings from the publications conducted in HICs has instigated the creation of educational stillbirth prevention campaigns in Australia, New Zealand and the United Kingdom [35]. These campaigns are targeted at pregnant women with a gestational age from 28 weeks with advice to modify going-to-sleep positions to adopt a left lateral sleep position.

Since our review identified only two studies conducted exclusively in LMICs, insufficient data was found to confirm the prevalence of maternal sleep positions. An observational study conducted in a low- and middle-income setting will be required to determine the prevalence of different maternal sleep positions among pregnant women from LMICs and if any positions are associated with an increased risk of stillbirth. Only once further research in this area is conducted can public health campaigns be created.

## Conclusion

This systematic review provides a summary of the available evidence regarding the prevalence of different maternal sleep positions during pregnancy and associated risk of stillbirth in LMICs. There is limited evidence available and as such the prevalence of sleep positions and associated stillbirth risk in LMICs remains inconclusive, given the lack of robustly conducted studies in these settings. Given supine going-to-sleep position has been found to be associated with an increased risk of late stillbirth in high-income settings, this suggests that sleep position may be a modifiable risk factor for stillbirth in LMICs. However, there is a need for further research to understand the sleep practices and behaviours of pregnant women in LMICs before public health initiatives can be developed and implemented.

## Supplementary Information

**Additional file 1.** PRISMA checklist.

**Additional file 2.** Search strategy.

**Additional file 3.** Data extraction tool.

## Data Availability

Not applicable.

## Abbreviations

AOR: Adjusted odds ratio
CI: Confidence interval
HIC: High-income country
IPD: Individual participant data
LMIC: Low- and middle-income country
NOS: Newcastle–Ottawa Scale
OR: Unadjusted odds ratio
