# Supplementary material for: Women’s sleep position during pregnancy in low- and middle-income countries: a systematic review
Source: Reprod Health. 2021 Mar 1;18:53. doi: 10.1186/s12978-021-01106-x (PMC7923448; doi:10.1186/s12978-021-01106-x)
Supplement: Supplementary file 2 — Additional file 2. Search strategy. [file 12978_2021_1106_MOESM2_ESM.pdf]

Appendix 2 Search Strategy

A.1.1 Ovid Medline search strategy

Database(s): **Ovid MEDLINE® and Epub Ahead of Print, In-Process & Other Non-Indexed Citations, Daily and Versions(R)** 1946 to April 02, 2020
Search date: 04 April 2020

Search Strategy:

#	Searches	Results
1	exp Pregnancy/	884,967
2	(pregnan* or maternal or gestation* or antenatal or prenatal or perinatal or natal or gravidit* or gravida* or multigravid* or primigravid*).mp.	1,207,647
3	(parity or parous or primiparity or multiparity or primiparous or multiparous).mp.	60,304
4	(expectant adj (mother* or wom#n or female*)).mp.	1,304
5	1 or 2 or 3 or 4	1,234,652
6	(sleep* or slept or lying down or laid down or lie down or lay down or recumben* or reclining or reclined).mp.	212,880
7	((supine or prone) adj3 (position* or posture*)).mp.	24,056
8	((lying or rest or rested or resting) and (position* or posture*)).mp.	18,718
9	6 or 7 or 8	248,516
10	5 and 9	9,426
11	Developing Countries/	74,062
12	(Afghanistan* or Albania* or Algeria* or Angola* or Argentina* or Armenia* or Azerbaijan* or Bangladesh* or Belarus* or Beliz* or Benin* or Bhutan* or Bolivia* or Bosnia* or Herzegovin* or Botswan* or Brazil* or Bulgaria* or Burkina* or Burundi* or Cabo Verde* or Cape Verde* or Cambodia* or Cameroon* or Central African or Chad* or China or Chinese or Colombia* or Comor* or Congo* or Costa Rica* or Cote d'Ivoir* or Ivory coast or Cuba* or Djibouti* or Dominica* or Ecuador* or Egypt* or El Salvador* or Eritrea* or Ethiopia* or Fiji* or Gabon* or Gambia* or Georgia* or Ghana* or Grenad* or Guatemala* or Guinea* or Guyan* or Haiti* or Hondura* or Hungar* or India* or Indonesia* or Iran* or Iraq* or Jamaica* or Jordan* or Kazakhstan* or Kenya* or Kiribati* or Korea* or Kosov* or Kyrgyz Republic or Lao* or Leban* or Lesotho* or Liberia* or Libya* or Macedonia* or Madagascar* or Malawi* or Malaysia* or Maldiv* or Mali* or Marshall Island* or Mauritania* or Mauriti* or Mexic* or Micronesia* or Moldova* or Mongolia* or Montenegr* or Morocc* or Mozambi* or Myanma* or Burmese or Namibia* or Nepal* or Nicaragua* or Niger* or Nigeria* or Pakistan* or Palau* or Panama* or Papua New Guinea* or Paraguay* or Peru* or Philippines or Filipino or Romania* or Rwanda* or Samoa* or	2,646,441

	Sao Tome* or Senegal* or Serbia* or Seychell* or Sierra Leon* or Soloman Island* or Somalia* or South Africa* or Sudan* or Sri Lanka* or St Lucia* or St Vincent or Grenadines or Surinam* or Swazi* or Syria* or Tajikistan* or Tanzania* or Thai* or Timor* or Togo* or Tonga* or Tunisia* or Turk* or Turkmenistan* or Tuvalu* or Uganda* or Ukrain* or Uzbekistan* or Vanuatu* or Venezuela* or Vietnam* or West Bank or Gaza or Yemen* or Zambia* or Zimbabwe*).mp.	
13	exp africa/ or exp caribbean region/ or exp central america/ or latin america/ or exp south america/ or asia/ or exp asia, central/ or exp asia, southeastern/ or exp asia, western/ or exp indian ocean islands/ or pacific islands/ or exp melanesia/ or exp micronesia/ or exp west indies/	845,891
14	(africa* or asia* or caribbean or central america* or latin america* or south america* or melanesia* or micronesia* or polynesia*).mp.	557,464
15	(resource-limit* or resource-poor or low-resource* or limited-resource* or resource-constrain* or constrain*-resource* or under-resource* or poor*-resource* or resource-scarce* or scarce*-resource* or low-income or middle-income or lowincome or middleincome or (low adj3 middle-income)).mp.	83,416
16	((developing or underdeveloped or under-developed or emerging or less-developed or least-developed or less-economically developed or least-economically developed or less-affluent or least-affluent or deprived or poor) adj (country or countries or nation or nations or region or regions or economy or economies)).mp.	131,753
17	((developing or underdeveloped or under-developed or less-developed or least-developed) adj (population* or world)).mp.	9,050
18	(third-world* or thirdworld* or 3rd-world* or lmic or lmics or lami countr* or lalmi countr* or transitional countr*).mp.	8,007
19	(low* adj (gdp or gnp or gross domestic or gross national)).mp.	239
20	((underserved or under served) adj (countr* or nation? or population*)).mp.	3,057
21	11 or 12 or 13 or 14 or 15 or 16 or 17 or 18 or 19 or 20	3,084,106
22	10 and 21	1,374
23	exp animals/ not humans.sh.	4,685,426
24	22 not 23	1,297
25	Exp Pregnancy, Animal/	25,449
26	24 not 25	1,297

A.1.2 Ovid Embase search strategy

Database(s): **Embase Classic+Embase** 1947 to 2020 April 02
Search date: 04 April 2020

Search Strategy:

#	Searches	Results
1	exp pregnancy/	795,893
2	(pregnan* or maternal or gestation* or antenatal or prenatal or perinatal or natal or gravidit* or gravida* or multigravid* or primigravid*).mp.	1,492,153

3	(parity or parous or primiparity or multiparity or primiparous or multiparous).mp.	72,777
4	(expectant adj (mother* or wom#n or female*)).mp.	1,894
5	1 or 2 or 3 or 4	1,518,394
6	(sleep* or slept or lying down or laid down or lie down or lay down or recumben* or reclining or reclined).mp.	358,311
7	((supine or prone) adj3 (position* or posture*)).mp.	41,080
8	((lying or rest or rested or resting) and (position* or posture*)).mp.	28,155
9	6 or 7 or 8	414,788
10	5 and 9	16,219
11	exp developing country/	95,379
12	(Afghanistan* or Albania* or Algeria* or Angola* or Argentina* or Armenia* or Azerbaijan* or Bangladesh* or Belarus* or Beliz* or Benin* or Bhutan* or Bolivia* or Bosnia* or Herzegovin* or Botswan* or Brazil* or Bulgaria* or Burkina* or Burundi* or Cabo Verde* or Cape Verde* or Cambodia* or Cameroon* or Central African or Chad* or China or Chinese or Colombia* or Comor* or Congo* or Costa Rica* or Cote d'Ivoir* or Ivory coast or Cuba* or Djibouti* or Dominica* or Ecuador* or Egypt* or El Salvador* or Eritrea* or Ethiopia* or Fiji* or Gabon* or Gambia* or Georgia* or Ghana* or Grenad* or Guatemala* or Guinea* or Guyan* or Haiti* or Hondura* or Hungar* or India* or Indonesia* or Iran* or Iraq* or Jamaica* or Jordan* or Kazakhstan* or Kenya* or Kiribati* or Korea* or Kosov* or Kyrgyz Republic or Lao* or Leban* or Lesotho* or Liberia* or Libya* or Macedonia* or Madagascar* or Malawi* or Malaysia* or Maldiv* or Mali* or Marshall Island* or Mauritania* or Mauriti* or Mexic* or Micronesia* or Moldova* or Mongolia* or Montenegr* or Morocc* or Mozambi* or Myanma* or Burmese or Namibia* or Nepal* or Nicaragua* or Niger* or Nigeria* or Pakistan* or Palau* or Panama* or Papua New Guinea* or Paraguay* or Peru* or Philippines or Filipino or Romania* or Rwanda* or Samoa* or Sao Tome* or Senegal* or Serbia* or Seychell* or Sierra Leon* or Soloman Island* or Somalia* or South Africa* or Sudan* or Sri Lanka* or St Lucia* or St Vincent or Grenadines or Surinam* or Swazi* or Syria* or Tajikistan* or Tanzania* or Thai* or Timor* or Togo* or Tonga* or Tunisia* or Turk* or Turkmenistan* or Tuvalu* or Uganda* or Ukrain* or Uzbekistan* or Vanuatu* or Venezuela* or Vietnam* or West Bank or Gaza or Yemen* or Zambia* or Zimbabwe*).mp.	3,750,176
13	exp Africa/ or exp caribbean/ or exp caribbean islands/ or exp South America/ or exp Central America/ or exp Asia/ or exp Indian ocean/ or exp pacific ocean/	1,640,698

14	(africa* or asia* or caribbean or central america* or latin america* or south america* or melanesia* or micronesia* or polynesia*).mp.	709,458
15	(resource-limit* or resource-poor or low-resource* or limited-resource* or resource-constrain* or constrain*-resource* or under-resource* or poor*-resource* or resource-scarce* or scarce*-resource* or low-income or middle-income or lowincome or middleincome or (low adj3 middle-income)).mp.	106,734
16	((developing or underdeveloped or under-developed or emerging or less-developed or least-developed or less-economically developed or least-economically developed or less-affluent or least-affluent or deprived or poor) adj (country or countries or nation or nations or region or regions or economy or economies)).mp.	147,288
17	((developing or underdeveloped or under-developed or less-developed or least-developed) adj (population* or world)).mp.	11,411
18	(third-world* or thirdworld* or 3rd-world* or lmic or lmics or lami countr* or lalmi countr* or transitional countr*).mp.	9,677
19	(low* adj (gdp or gnp or gross domestic or gross national)).mp.	346
20	((underserved or under served) adj (countr* or nation? or population*)).mp.	4,543
21	11 or 12 or 13 or 14 or 15 or 16 or 17 or 18 or 19 or 20	4,538,802
22	10 and 21	2,828
23	exp breeding/	50,299
24	22 not 23	2,824
25	(exp animal/ or nonhuman/) not exp human/	7,135,403
26	24 not 25	2,700

A.1.3 CINAHL search strategy

CINAHL Complete
Search date: 04 April 2020

Search strategy:

#	Query	Results
S1	(MH "Pregnancy+")	221,733
S2	((pregnan* or maternal or gestation* or antenatal or prenatal or perinatal or natal or gravidit* or gravida* or multigravid* or primigravid*)) OR ((parity or parous or primiparity or multiparity or primiparous or multiparous)) OR ((expectant adj (mother* or wom#n or female*)))	339,026
S3	S1 or S2	345,904
S4	(sleep* or slept or lying down or laid down or lie down or lay down or recumben* or reclining or reclined)	78,377
S5	((supine or prone) N3 (position* or posture*))	7,990
S6	((lying or rest or rested or resting) and (position* or posture*))	4,216
S7	S4 or S5 or S6	88,178
S8	S3 and S7	4,146
S9	(MH "Developing Countries")	19,300
S10	(Afghanistan* or Albania* or Algeria* or Angola* or Argentina* or Armenia* or Azerbaijan* or Bangladesh* or Belarus* or Beliz* or	697,038

	Benin* or Bhutan* or Bolivia* or Bosnia* or Herzegovin* or Botswan* or Brazil* or Bulgaria* or Burkina* or Burundi* or Cabo Verde* or Cape Verde* or Cambodia* or Cameroon* or Central African or Chad* or China or Chinese or Colombia* or Comor* or Congo* or Costa Rica* or Cote d'Ivoir* or Ivory coast or Cuba* or Djibouti* or Dominica* or Ecuador* or Egypt* or El Salvador* or Eritrea* or Ethiopia* or Fiji* or Gabon* or Gambia* or Georgia* or Ghana* or Grenad* or Guatemala* or Guinea* or Guyan* or Haiti* or Hondura* or Hungar* or India* or Indonesia* or Iran* or Iraq* or Jamaica* or Jordan* or Kazakhstan* or Kenya* or Kiribati* or Korea* or Kosov* or Kyrgyz Republic or Lao* or Leban* or Lesotho* or Liberia* or Libya* or Macedonia* or Madagascar* or Malawi* or Malaysia* or Maldiv* or Mali* or Marshall Island* or Mauritania* or Mauriti* or Mexic* or Micronesia* or Moldova* or Mongolia* or Montenegr* or Morocc* or Mozambi* or Myanma* or Burmese or Namibia* or Nepal* or Nicaragua* or Niger* or Nigeria* or Pakistan* or Palau* or Panama* or Papua New Guinea* or Paraguay* or Peru* or Philippines or Filipino or Romania* or Rwanda* or Samoa* or Sao Tome* or Senegal* or Serbia* or Seychell* or Sierra Leon* or Soloman Island* or Somalia* or South Africa* or Sudan* or Sri Lanka* or St Lucia* or St Vincent or Grenadines or Surinam* or Swazi* or Syria* or Tajikistan* or Tanzania* or Thai* or Timor* or Togo* or Tonga* or Tunisia* or Turk* or Turkmenistan* or Tuvalu* or Uganda* or Ukrain* or Uzbekistan* or Vanuatu* or Venezuela* or Vietnam* or West Bank or Gaza or Yemen* or Zambia* or Zimbabwe*)	
S11	(MH ”Africa+”) or (MH ”Latin America”) or (MH “Central America+”) or (MH “South America+”) or (MH “West Indies+”) or (MH “Asia+”) or (MH “Atlantic Islands+”) or (MH “Indian Ocean Islands+”) or (MH “Melanesia+”) or (MH “Micronesia+”) or (MH “Samoa+”)	462,910
S12	(africa* or asia* or caribbean or central america* or latin america* or south america* or melanesia* or micronesia* or polynesia*)	149,335
S13	(resource-limit* or resource-poor or low-resource* or limited-resource* or resource-constrain* or constrain*-resource* or under-resource* or poor*-resource* or resource-scarce* or scarce*-resource* or low-income or middle-income or lowincome or middleincome or (low N3 middle-income))	42,073
S14	(developing or underdeveloped or under-developed or emerging or less-developed or least-developed or less-economically developed or least-economically developed or less-affluent or least-affluent or deprived or poor) N1 (country or countries or nation or nations or region or regions or economy or economies)	32,103
S15	(developing or underdeveloped or under-developed or less-developed or least-developed) N1 (population* or world)	2,823
S16	(third-world* or thirdworld* or 3rd-world* or lmic or lmics or lami countr* or lalmi countr* or transitional countr*) OR ((low* N1 (gdp or gnp or gross domestic or gross national)))	8,167
S17	((underserved or under served) N1 (countr* or nation? or population*))	2,321
S18	S9 OR S10 OR S11 OR S12 OR S13 OR S14 OR S15 OR S16 OR S17	894,170
S19	S8 and S18	807

A.1.4 Ovid Emcare search strategy

Database(s): **Ovid Emcare** 1995 to 2020 week 13
Search date: 04 April 2020

Search Strategy:

#	Searches	Results
1	exp pregnancy/	117,279
2	(pregnan* or maternal or gestation* or antenatal or prenatal or perinatal or natal or gravidit* or gravida* or multigravid* or primigravid*).mp.	276,407
3	(parity or parous or primiparity or multiparity or primiparous or multiparous).mp.	14,959
4	(expectant adj (mother* or wom#n or female*)).mp.	626
5	1 or 2 or 3 or 4	281,390
6	(sleep* or slept or lying down or laid down or lie down or lay down or recumben* or reclining or reclined).mp.	77,544
7	((supine or prone) adj3 (position* or posture*)).mp.	11,584
8	((lying or rest or rested or resting) and (position* or posture*)).mp.	5,785
9	6 or 7 or 8	91,650
10	5 and 9	4,392
11	developing countr*.mp.	29,445
12	(Afghanistan* or Albania* or Algeria* or Angola* or Argentina* or Armenia* or Azerbaijan* or Bangladesh* or Belarus* or Beliz* or Benin* or Bhutan* or Bolivia* or Bosnia* or Herzegovin* or Botswan* or Brazil* or Bulgaria* or Burkina* or Burundi* or Cabo Verde* or Cape Verde* or Cambodia* or Cameroon* or Central African or Chad* or China or Chinese or Colombia* or Comor* or Congo* or Costa Rica* or Cote d'Ivoir* or Ivory coast or Cuba* or Djibouti* or Dominica* or Ecuador* or Egypt* or El Salvador* or Eritrea* or Ethiopia* or Fiji* or Gabon* or Gambia* or Georgia* or Ghana* or Grenad* or Guatemala* or Guinea* or Guyan* or Haiti* or Hondura* or Hungar* or India* or Indonesia* or Iran* or Iraq* or Jamaica* or Jordan* or Kazakhstan* or Kenya* or Kiribati* or Korea* or Kosov* or Kyrgyz Republic or Lao* or Leban* or Lesotho* or Liberia* or Libya* or Macedonia* or Madagascar* or Malawi* or Malaysia* or Maldiv* or Mali* or Marshall Island* or Mauritania* or Mauriti* or Mexic* or Micronesia* or Moldova* or Mongolia* or Montenegr* or Morocc* or Mozambi* or Myanma* or Burmese or Namibia* or Nepal* or Nicaragua* or Niger* or Nigeria* or Pakistan* or Palau* or Panama* or Papua New Guinea* or Paraguay* or Peru* or Philippines or Filipino or Romania* or Rwanda* or Samoa* or Sao Tome* or Senegal* or Serbia* or Seychell* or Sierra Leon* or Soloman Island* or Somalia* or South Africa* or Sudan* or Sri Lanka* or St Lucia* or St Vincent or Grenadines or Surinam* or Swazi* or Syria* or Tajikistan* or Tanzania* or Thai* or Timor* or Togo* or Tonga* or Tunisia* or Turk* or Turkmenistan* or Tuvalu* or Uganda* or Ukrain* or Uzbekistan* or Vanuatu* or Venezuela* or Vietnam* or West Bank or Gaza or Yemen* or Zambia* or Zimbabwe*)	690,580
13	(africa* or asia* or caribbean or central america* or latin america* or south america* or melanesia* or micronesia* or polynesia*).mp.	151,429

14	(resource-limit* or resource-poor or low-resource* or limited-resource* or resource-constrain* or constrain*-resource* or under-resource* or poor*-resource* or resource-scarce* or scarce*-resource* or low-income or middle-income or lowincome or middleincome or (low adj3 middle-income)).mp.	45,093
15	((developing or underdeveloped or under-developed or emerging or less-developed or least-developed or less-economically developed or least-economically developed or less-affluent or least-affluent or deprived or poor) adj (country or countries or nation or nations or region or regions or economy or economies)).mp.	32,159
16	((developing or underdeveloped or under-developed or less-developed or least-developed) adj (population* or world)).mp.	3,228
17	(third-world* or thirdworld* or 3rd-world* or lmic or lmics or lami countr* or lalmi countr* or transitional countr*).mp.	3,090
18	(low* adj (gdp or gnp or gross domestic or gross national)).mp.	72
19	((underserved or under served) adj (countr* or nation? or population*)).mp.	1,931
20	11 or 12 or 13 or 14 or 15 or 16 or 17 or 18 or 19	811,037
21	10 and 20	778
22	(exp animal/ or nonhuman/) not exp human/	442,079
23	21 not 22	775

A.1.5 Global Medicus Index search strategy

Search date: 04 April 2020

Search Strategy:

**Search**	**Results**
(“pregnancy” or “pregnant” or “maternal” or “gestation” or “parity” or “parturition” or “gravidity”)<br>AND<br>(“sleep” or “slept” or “sleeping” or “rest” or “lying down” or “laid down” or “sleeping position” or “sleeping posture”)	154
