# Supplementary material for: Women’s sleep position during pregnancy in low- and middle-income countries: a systematic review
Source: Reprod Health. 2021 Mar 1;18:53. doi: 10.1186/s12978-021-01106-x (PMC7923448; doi:10.1186/s12978-021-01106-x)
Supplement: Supplementary file 3 — Additional file 3. Data extraction tool. [file 12978_2021_1106_MOESM3_ESM.pdf]

Appendix 3 Data extraction tool

The data extraction tool was used to assist in extracting relevant data from eligible studies. Both reviewers used this tool.

1. **Identifier and article information**
   - **1.1** Name of person extracting the data
   - **1.2** Date of data extraction
   - **1.3** Article identifier
   - **1.4** Article title
   - **1.5** First author's last name
   - **1.6** Year of publication
   - **1.7** Publication type
   - **1.8** Funding source

2. **Study design information**
   - **2.1** Aim of the study
   - **2.2** Study design
   - **2.3** Type of observational study
   - **2.4** Study location
   - **2.5** Setting of the study
   - **2.6** Population description
   - **2.7** Methods of recruitment of participants
   - **2.8** Sampling technique
   - **2.9** Start date
   - **2.10** End date
   - **2.11** Duration of study

3. **Demographic information**
   - **3.1** Sample size/total number of participants
   - **3.2** Ethnicity of the study population
   - **3.3** Age of youngest participant
   - **3.4** Age of eldest participant
   - **3.5** Average age of participants
   - **3.6** Age standard deviation of participants
   - **3.7** Are the participants pregnant women?

3.7.1 Number of weeks pregnant

3.7.2 Minimum number of weeks pregnant

3.7.3 Maximum number of weeks pregnant

3.8 Are the participants postpartum women?

3.8.1 Minimum number of hours postpartum

3.8.2 Maximum number of hours postpartum

3.8.3 Minimum number of weeks postpartum

3.8.4 Maximum number of weeks postpartum

3.9 Number of women in study population who were nulliparous

3.10 Percentage of women in study population who were nulliparous

3.11 Number of women in study population who had a parity of 1-2

3.12 Percentage of women in study population who had a parity of 1-2

3.13 Number of women in study population who had a parity of 3-4

3.14 Percentage of women in study population who had a parity of 3-4

3.15 Number of women in study population who had a parity of ≥5

3.16 Percentage of women in study population who had a parity of ≥5

4. Primary outcome

4.1 Was the prevalence of supine sleep position during pregnancy reported?

4.1.1 Description of supine sleep position during pregnancy

4.1.2 Collection of maternal supine sleep position information

4.1.3 Number of women in study population who reported supine sleep position during pregnancy

4.1.4 Number of women in study population who gave sleep position information

4.1.5 Percentage of women in study population who reported supine sleep position during pregnancy

4.2 Was the prevalence of non-supine sleep position during pregnancy reported?

4.2.1 Description of non-supine sleep position during pregnancy

4.2.2 Collection of maternal non-supine sleep position information

4.2.3 Number of women in study population who reported non-supine sleep position during pregnancy

4.2.4 Number of women in study population who gave sleep position information

4.2.5 Percentage of women in study population who reported non-supine sleep position during pregnancy

4.3 Was the prevalence of prone sleep position during pregnancy reported?

- **4.3.1** Description of prone sleep position during pregnancy
- **4.3.2** Collection of maternal prone sleep position information
- **4.3.3** Number of women in study population who reported prone sleep position during pregnancy
- **4.3.4** Number of women in study population who gave sleep position information
- **4.3.5** Percentage of women in study population who reported prone sleep position during pregnancy

4.4 Was the prevalence of non-prone sleep position during pregnancy reported?

- **4.4.1** Description of non-prone sleep position during pregnancy
- **4.4.2** Collection of maternal non-prone sleep position information
- **4.4.3** Number of women in study population who reported non-prone sleep position during pregnancy
- **4.4.4** Number of women in study population who gave sleep position information
- **4.4.5** Percentage of women in study population who reported non-prone sleep position during pregnancy

4.5 Was the prevalence of lateral sleep position during pregnancy reported?

- **4.5.1** Description of lateral sleep position during pregnancy
- **4.5.2** Collection of maternal lateral sleep position information
- **4.5.3** Number of women in study population who reported lateral sleep position during pregnancy
- **4.5.4** Number of women in study population who gave sleep position information
- **4.5.5** Percentage of women in study population who reported lateral sleep position during pregnancy

4.6 Was the prevalence of non-lateral sleep position during pregnancy reported?

- **4.6.1** Description of non-lateral sleep position during pregnancy
- **4.6.2** Collection of maternal non-lateral sleep position information
- **4.6.3** Number of women in study population who reported non-lateral sleep position during pregnancy
- **4.6.4** Number of women in study population who gave sleep position information
- **4.6.5** Percentage of women in study population who reported non-lateral sleep position during pregnancy

4.7 Was the prevalence of left lateral sleep position during pregnancy reported?

- **4.7.1** Description of left lateral sleep position during pregnancy

4.7.2 Collection of maternal left lateral sleep position information

4.7.3 Number of women in study population who reported left lateral sleep position during pregnancy

4.7.4 Number of women in study population who gave sleep position information

4.7.5 Percentage of women in study population who reported left lateral sleep position during pregnancy

4.8 Was the prevalence of non-left lateral sleep position during pregnancy reported?

4.8.1 Description of non-left lateral sleep position during pregnancy

4.8.2 Collection of maternal non-left lateral sleep position information

4.8.3 Number of women in study population who reported non-left lateral sleep position during pregnancy

4.8.4 Number of women in study population who gave sleep position information

4.8.5 Percentage of women in study population who reported non-left lateral sleep position during pregnancy

4.9 Was the prevalence of right lateral sleep position during pregnancy reported?

4.9.1 Description of right lateral sleep position during pregnancy

4.9.2 Collection of maternal right lateral sleep position information

4.9.3 Number of women in study population who reported right lateral sleep position during pregnancy

4.9.4 Number of women in study population who gave sleep position information

4.9.5 Percentage of women in study population who reported right lateral sleep position during pregnancy

4.10 Was the prevalence of non-right lateral sleep position during pregnancy reported?

4.10.1 Description of non-right lateral sleep position during pregnancy

4.10.2 Collection of maternal non-right lateral sleep position information

4.10.3 Number of women in study population who reported non-right lateral sleep position during pregnancy

4.10.4 Number of women in study population who gave sleep position information

4.10.5 Percentage of women in study population who reported non-right lateral sleep position during pregnancy

5. Secondary outcome – prevalence of stillbirth

5.1 Was the prevalence of stillbirth reported?

5.1.1 Collection of stillbirth information

5.1.2 Number of women in study population who had a stillbirth

5.1.3 Number of women in study population who gave stillbirth information

5.1.4 Percentage of women in study population who had a stillbirth

6. Secondary outcome – measure of association between maternal sleep position during pregnancy and stillbirth

6.1 Was a measure of association between maternal supine sleep position during pregnancy and stillbirth reported?

6.1.1 Statistical method used

6.1.2 Type of measure of association

6.1.3 Result for measure of association

6.1.4 Lower limit of the 95% confidence interval for measure of association

6.1.5 Upper limit of the 95% confidence interval for measure of association

6.1.6 P-value for measure of association

6.1.7 Response/non-response rate for measure of association

6.2 Was a measure of association between maternal non-supine sleep position during pregnancy and stillbirth reported?

6.2.1 Statistical method used

6.2.2 Type of measure of association

6.2.3 Result for measure of association

6.2.4 Lower limit of the 95% confidence interval for measure of association

6.2.5 Upper limit of the 95% confidence interval for measure of association

6.2.6 P-value for measure of association

6.2.7 Response/non-response rate for measure of association

6.3 Was a measure of association between maternal prone sleep position during pregnancy and stillbirth reported?

6.3.1 Statistical method used

6.3.2 Type of measure of association

6.3.3 Result for measure of association

6.3.4 Lower limit of the 95% confidence interval for measure of association

6.3.5 Upper limit of the 95% confidence interval for measure of association

6.3.6 P-value for measure of association

6.3.7 Response/non-response rate for measure of association

6.4 Was a measure of association between maternal non-prone sleep position during pregnancy and stillbirth reported?

6.4.1 Statistical method used

6.4.2 Type of measure of association

6.4.3 Result for measure of association

6.4.4 Lower limit of the 95% confidence interval for measure of association

6.4.5 Upper limit of the 95% confidence interval for measure of association

6.4.6 P-value for measure of association

6.4.7 Response/non-response rate for measure of association

6.5 Was a measure of association between maternal lateral sleep position during pregnancy and stillbirth reported?

6.5.1 Statistical method used

6.5.2 Type of measure of association

6.5.3 Result for measure of association

6.5.4 Lower limit of the 95% confidence interval for measure of association

6.5.5 Upper limit of the 95% confidence interval for measure of association

6.5.6 P-value for measure of association

6.5.7 Response/non-response rate for measure of association

6.6 Was a measure of association between maternal non-lateral sleep position during pregnancy and stillbirth reported?

6.6.1 Statistical method used

6.6.2 Type of measure of association

6.6.3 Result for measure of association

6.6.4 Lower limit of the 95% confidence interval for measure of association

6.6.5 Upper limit of the 95% confidence interval for measure of association

6.6.6 P-value for measure of association

6.6.7 Response/non-response rate for measure of association

6.7 Was a measure of association between maternal left lateral sleep position during pregnancy and stillbirth reported?

6.7.1 Statistical method used

6.7.2 Type of measure of association

6.7.3 Result for measure of association

6.7.4 Lower limit of the 95% confidence interval for measure of association

6.7.5 Upper limit of the 95% confidence interval for measure of association

6.7.6 P-value for measure of association

6.7.7 Response/non-response rate for measure of association

6.8 Was a measure of association between maternal non-left lateral sleep position during pregnancy and stillbirth reported?

6.8.1 Statistical method used

6.8.2 Type of measure of association

6.8.3 Result for measure of association

6.8.4 Lower limit of the 95% confidence interval for measure of association

6.8.5 Upper limit of the 95% confidence interval for measure of association

6.8.6 P-value for measure of association

6.8.7 Response/non-response rate for measure of association

6.9 Was a measure of association between maternal right lateral sleep position during pregnancy and stillbirth reported?

6.9.1 Statistical method used

6.9.2 Type of measure of association

6.9.3 Result for measure of association

6.9.4 Lower limit of the 95% confidence interval for measure of association

6.9.5 Upper limit of the 95% confidence interval for measure of association

6.9.6 P-value for measure of association

6.9.7 Response/non-response rate for measure of association

6.10 Was a measure of association between maternal non-right lateral sleep position during pregnancy and stillbirth reported?

6.10.1 Statistical method used

6.10.2 Type of measure of association

6.10.3 Result for measure of association

6.10.4 Lower limit of the 95% confidence interval for measure of association

6.10.5 Upper limit of the 95% confidence interval for measure of association

6.10.6 P-value for measure of association

6.10.7 Response/non-response rate for measure of association

7. Conclusions

7.1 What were the key conclusions found by the authors?
